# Immune dysregulation in sepsis: experiences, lessons and perspectives

**DOI:** 10.1038/s41420-023-01766-7

**Published:** 2023-12-19

**Authors:** Min Cao, Guozheng Wang, Jianfeng Xie

**Affiliations:** 1 Jiangsu Provincial Key Laboratory of Critical Care Medicine, Department of Critical Care Medicine, Zhongda Hospital, School of Medicine, Southeast University, Nanjing, China; 2 Department of Clinical Infection, Microbiology and Immunology, University of Liverpool, Liverpool, L69 7BE UK; 3 Coagulation, Liverpool University Hospitals NHS Foundation Trust, Liverpool, L7 8XP UK

**Keywords:** Bacterial infection, Viral infection

## Abstract

Sepsis is a life-threatening organ dysfunction syndrome caused by dysregulated host responses to infection. Not only does sepsis pose a serious hazard to human health, but it also imposes a substantial economic burden on the healthcare system. The cornerstones of current treatment for sepsis remain source control, fluid resuscitation, and rapid administration of antibiotics, etc. To date, no drugs have been approved for treating sepsis, and most clinical trials of potential therapies have failed to reduce mortality. The immune response caused by the pathogen is complex, resulting in a dysregulated innate and adaptive immune response that, if not promptly controlled, can lead to excessive inflammation, immunosuppression, and failure to re-establish immune homeostasis. The impaired immune response in patients with sepsis and the potential immunotherapy to modulate the immune response causing excessive inflammation or enhancing immunity suggest the importance of demonstrating individualized therapy. Here, we review the immune dysfunction caused by sepsis, where immune cell production, effector cell function, and survival are directly affected during sepsis. In addition, we discuss potential immunotherapy in septic patients and highlight the need for precise treatment according to clinical and immune stratification.

## Facts

- Sepsis is a dynamic disorder of dysregulated inflammatory and immune responses.
- Heterogeneity is present in patients with sepsis.
- There are currently no effective therapeutic options available for sepsis in the clinic.
- Individualized immunotherapy based on the individual immunological characteristics of sepsis patients is a reasonable and promising therapeutic option.

## Open questions

- How to establish an early warning system for sepsis and find effective biomarkers and immune checkpoints regarding individual immunological characteristics of sepsis patients?
- How to clarify the mechanisms of immune cell dysfunction in sepsis for the understanding the personalized treatment of these heterogeneous and dynamic stages of sepsis?
- How to combine advanced technologies (such as multi-omics analysis and artificial intelligence) for prospective studies of personalized therapy in multiple clinical settings to improve model universality?

## Introduction

**Fig. 1:**
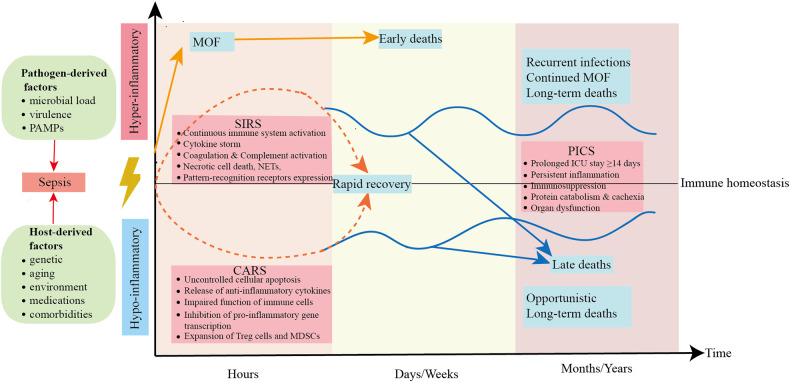
Host immune response in sepsis. Activation of both proinflammatory and anti‐inflammatory immune responses occurs promptly after sepsis onset. The host response to severe sepsis can have four different clinical trajectories: (1) early MOF leading to death, (2) rapid recovery, (3) late deaths, or (4) late sequelae or long-term deaths. SIRS, systemic inflammatory response syndrome; CARS compensatory anti‐inflammatory response syndrome, MOF multi-organ failure, NETs Neutrophil extracellular traps, MDSCs Myeloid-derived suppressor cells, ICU intensive care unit, PICS persistent inflammation, immunosuppression, and catabolism syndrome.

Sepsis is a life-threatening, complex clinical and biochemical syndrome characterized by acute organ dysfunction that develops due to the body’s dysfunctional response to microbial invasion [1]. Sepsis remains a significant cause of health loss worldwide, with an estimated 48.9 million incident cases of sepsis and 11 million sepsis-related deaths [2]. Our previous cross-sectional study revealed that sepsis impacted one-fifth of ICU-admitted patients and has a 90-day mortality rate of 35.5%, indicating a substantial burden of sepsis on the Chinese mainland [3]. The pathophysiology of sepsis is complex when the pathogen evades the host’s defense mechanisms and continuously stimulates and damages host cells so that many of the immune mechanisms initially activated to provide protection have become deleterious due to the inability to restore homeostasis, leading to persistent hyperinflammation and immunosuppression [4]. From the 1970s until the early 2000s, it was widely recognized that the high mortality rate in sepsis was caused by multiple organ failure due to immune damage resulting from an excessive inflammatory response. However, all anti-inflammatory therapy strategies were failed in clinical trials. In recent years, a substantial body of research has shown that sepsis is characterized by concurrent dysregulation of the innate immune system and suppression of the adaptive immune system. This simultaneous imbalance and persistence of inflammatory and anti-inflammatory responses ultimately culminate in recurrent and persistent infections, organ dysfunction, and ultimately, fatality for the patient (Fig. 1). Numerous individuals afflicted with sepsis may experience a comparatively concise phase of hyperinflammation, nonetheless are susceptible to developing immunocompromised states due to extended hospitalization and recuperation. Indeed, a significant proportion of individuals with sepsis die from secondary or opportunistic infections while in a condition of immunosuppression. Hence, it is imperative to ascertain the immune status of individuals with sepsis, elucidate the clinical and immunological categorization of patients, and effectively regulate the exaggerated inflammatory response and immunosuppressive condition of the septic patient’s body, all of which are crucial in the management of sepsis. Here, we review the key factors of immune dysregulation in sepsis and potential precision immunotherapies.

### Immune dysregulation in sepsis

**Fig. 2:**
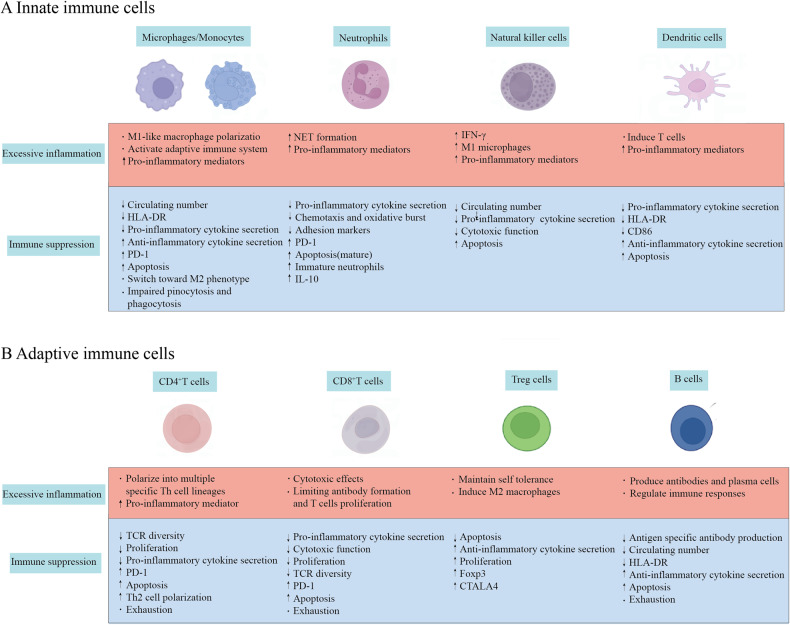
Sepsis-induced immune dysregulation. **A** Innate immune dysfunction in sepsis. **B** Adaptive immune dysfunction in sepsis. When septic insults happen, both the innate and adaptive immune responses are drastically changed. Shortly after detection of an infectious agent, the innate immune cells attempt to clear the overwhelming infection as quickly as possible, followed by activation of the adaptive immune system through activation of the Th cells and cytotoxic T cells. Under normal conditions, after the resolution of the infection, the patient’s body will return to homeostasis. In inflammatory responses due to severe infections, immune cells undergo various phenotypic changes as the immune system fails to resolve inflammation appropriately. Immune cell production, effector cell function, and survival are directly affected, resulting in ubiquitous immunosuppression. HLA-DR human leukocyte antigen-antigen D related, PD-1 programmed cell death protein 1, IL interleukin, TCR T-cell receptor.

Innate and adaptive immune cells activated by pathogen invasion relocate locally to tissues to prevent microbial multiplication and spread, and immunological homeostasis is attained when inflammation is controlled. During the onset of sepsis, pro-inflammatory and anti-inflammatory processes are activated simultaneously. Innate and adaptive immune cells triggered by both pathogens and DAMPs are in a state of hyperinflammation, and cytokine storms produced by immune cells block infections to some extent but also contribute to severe tissue damage. Pathogens can spread throughout the body through damaged blood vessels, causing an intense inflammatory response, leading to systemic immune dysregulation and injury. Depletion of innate and adaptive immune cells through apoptosis can lead to immunosuppression. Multiple factors influence the immune response in sepsis, including co-morbidities (e.g., malignancy, diabetes, heart disease), the microbial inoculum quantity, and the pathogen’s virulence. The principal pathogens causing sepsis include bacteria, fungi, and viruses. Superantigens of Gram-positive bacteria can cause significant direct harm to host cells. Lipopolysaccharides, the surface toxins of Gram-negative bacteria, can stimulate specific toll-like receptors, leading to a devastating immune response. Although the clinical appearance of viral sepsis is similar to that of bacterial sepsis, the immunological response is different. Macrophages boost the production of type I and type II interferons after exposure to the virus, and further activated neutrophils and lymphocytes play a critical role against the virus. Unlike other pathogens, fungal infections are usually connected with a situation of immunosuppression, and it is only after an immunological imbalance that the fungus invades deeper tissues, leading to sepsis. Fungal sepsis, hence, has a higher mortality rate compared to viral and bacterial sepsis. The progression of sepsis follows a certain pattern of immunodynamic change, with patients having distinct immune statuses at different times and stages, and the same immune cells presenting varied patterns of immune status. Innate and adaptive immune responses are considerably altered during the development of sepsis, which may impair the host’s ability to destroy invading pathogens, further leading to the recurrence of latent infections and susceptibility to secondary infections (Fig. 2).

During early sepsis, macrophages’ toll-like receptor 4 (TLR4) recognizes LPS, activating the nuclear factor-κB (NF-κB) and mitogen-activated protein kinase (MAPK) pathways to release proinflammatory cytokines and clear pathogenic microorganisms [5]. Meanwhile, massive apoptosis of macrophages and secretion of large amounts of anti-inflammatory mediators by M2-like macrophages make it difficult for the host to respond effectively to the pathogen. Excessive neutrophil activation and neutrophil extracellular traps (NETs) release may induce a shift in endothelial cells toward a proinflammatory and procoagulant phenotype and macrophage polarization toward the M1 phenotype [6, 7]. However, pro-inflammatory cytokines can upregulate guanosine cerebrospinal fluid levels, which can lead to an excessive release of circulating immature neutrophils [8]. NK cells activation is dysregulated and secretes large amounts of cytokines, contributing to a positive feedback loop and amplifying the pro-inflammatory cytokine storm [9]. However, the number of NK cells, cytokine production, and cytotoxic proteins from NK cells are decreased during the immunosuppressive stage of sepsis. Sepsis also can lead to apoptosis of dendritic cells(DCs) cells, block their maturation process and induce paralysis to reduce the number of DCs [10]. In addition, the levels of surface molecules associated with the function of DCs are changed, leading to immune tolerance.

Numbers of CD4 T cells decrease after the onset of sepsis, and absolute CD4 T-cell numbers return to pre-septic levels after a month in most patients, but failure to restore sufficient numbers of immunocompetent CD4 T cells is associated with a poor prognosis [11, 12]. Impaired CD4 T cell function after sepsis, characterized by decreased cytokine secretion and increased expression of inhibitory receptors, restricts the assistance provided to other immune cells [13]. Sepsis also disrupts the expression and function of CD4T cell subsets (Th1, Th2, Th17 and Treg subsets). Our previous study showed that Th2/Th1 values were significantly upregulated in previously immunocompetent patients at the onset of community-acquired severe sepsis, and their sustained dynamic increase was associated with ICU-acquired infection and 28-day mortality [14]. The Th17/Treg balance is regarded as a key factor in the homeostasis of the internal immune environment, and imbalance has been shown to be associated with the aggravation of illness in patients with sepsis [15]. Additionally, the composition and phenotype of the circulating CD8 T cell pool are altered after sepsis, inducing a rapid loss of naïve CD8 T cells and memory CD8 T cells leading to transient lymphocytopenia with early signs of immune paralysis [16]. B-cell number, phenotype, and effector functions are also significantly altered in sepsis patients and are inconsistent across populations [17]. Our current understanding of immune cells in the development of sepsis remains limited, but scientific advances continue to fill critical knowledge gaps and are also gradually identifying new potential therapeutic targets.

### Immunotherapy

**Fig. 3:**
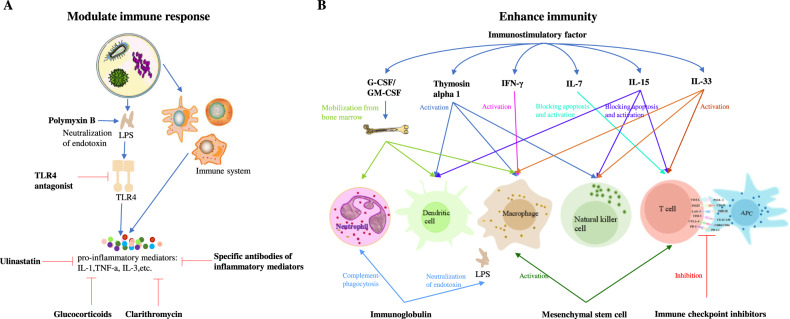
Potential immunotherapy for patients with sepsis-modulate the immune responses or enhance immunity. **A** Modulate immune responses that provoke excessive inflammation during sepsis. **B** Enhance immunity during sepsis. The immune response in sepsis is a highly individualized process. Sepsis patients’ immune responses vary depending on their immunological condition at the time of infection, age, comorbidities, environmental variables, and microbiome. Precise immunotherapy can significantly improve the prognosis of sepsis. LPS lipopolysaccharide, TLR4 toll like receptor 4, IL interleukin, TNF-α tumor necrosis factor-alpha, G-CSF Granulocyte colony-stimulating factor, GM-CSF granulocyte macrophage colony stimulating factor, IFN-γ interferon-gamma, APC antigen-presenting cell.

Imbalance of the immune system in sepsis patients is one of the main causes of their poor prognosis. The pathogenesis of sepsis includes not only excessive inflammatory response, but also a number of molecular and cellular events that contribute to immunosuppression. Numerous clinical studies on immunotherapy have focused on how to modulate immune response and enhance immunity in patients with sepsis (Supplemental Table 1). Restoring immune function and immune balance can reduce harm to organ function in patients with immune imbalances, protecting the organs.The regulatory effect on the immune-response modulation is primarily concerned with the inflammatory response, i.e. the balancing and regulating effect on the inflammatory response. Immunity enhancement can further maintain the immune homeostasis of the organism through the reconstruction of immune function. Many patients with sepsis have a relatively brief phase of hyperinflammation, so the success of drugs targeting inflammation may be effective only for a very short period of time. Patients with sepsis may also become immunocompromised, so a “one-size-fits-all” treatment strategy for sepsis-induced immune imbalance is bound to fail. In general, precise regulation of the excessive inflammatory response and immunosuppressive state of the body in sepsis patients is the key to treating sepsis (Fig. 3).

### Modulate immune response

#### Specific antibodies of inflammatory mediators

##### IL-1

IL-1ra administration in late sepsis decreases hypothalamic oxidative stress and increases vasopressin production, enhancing blood pressure and animal survival [18]. Knaus et al. found that patients with sepsis treated with recombinant human (rh)IL-1ra had a significant increase in survival time [19]. However, a further retrospective analysis showed that survival was not statistically significant in all patients treated with rIL-1ra compared with the placebo group [20]. The study by Opal et al. was terminated after an interim analysis found that a 72-hour continuous intravenous infusion of anakinra(rIL-1ra) failed to reduce mortality statistically significantly [21]. A reanalysis by Shakoory et al. found that anakinra significantly improved survival in patients with sepsis complicated by hepatobiliary dysfunction and disseminated intravascular coagulation (HBD/DIC) [22]. Based on early soluble urokinase plasminogen activator receptor(suPAR), subcutaneous anakinra reduced severe respiratory failure and restored the pro/anti-inflammatory balance [23]. The PROVIDE trial defined a rapid classification of sepsis from an immunological perspective in patients with macrophage activation-like syndrome (mALS), or immune paralysis who were randomly assigned to anakinra or rhIFNγ or placebo treatment groups, with 42.9% of patients surviving after 7 days with a decrease in sequential organ failure assessment (SOFA) score [24]. In the ongoing clinical trial (NCT04990232), based on the measurement of circulating ferritin and HLA-DR expression, patients were classified as hyper-inflammatory or immunoparalysis and were randomly assigned to either a placebo arm or an immunotherapy arm(anakinra or rhIFN-γ) [25]. The above studies show that treatment with anakinra in personalized adjuvant immunotherapy is promising but should be confirmed with more optimized trial protocols in future studies. The above studies suggest that personalized adjuvant immunotherapy after determining a patient’s immunophenotype will hopefully benefit sepsis patients.

#### TNF-α

Neutralizing monoclonal anti-cachectin/TNFα monoclonal antibodies injected only one hour before a bacterial attack in baboons prevented shock, while two hours prevented essential organ dysfunction and mortality [26]. In patients with severe sepsis or septic shock, high doses of the murine anti-TNF-α antibody, CB0006, were well tolerated, with a tendency to improve survival in the subgroup with high plasma TNF-α concentrations [27]. Polyclonal ovine anti-TNF-α fragment antigen binding (Fab) fragments (CytoFab) on plasma TNF-α were effectively reducing serum and BAL TNF-α and serum IL-6 concentrations, and increasing the number of ventilator-free and ICU-free days at day 28 [28]. Reinhart et al. retrospectively stratified severe sepsis or septic shock patients based on IL-6 concentrations and showed that MAK 195 F reduced mortality in patients with baseline IL-6 concentrations above 1000 pg/mL [29]. However, the subsequent study of sepsis patients with IL-6 concentrations >1000 pg/mL who were randomly assigned to receive afeliomab or placebo was terminated early after the primary efficacy endpoint was estimated not to be met due to interim analysis [30]. More and more research is showing that immune dysregulation in sepsis cannot be attributed to a single cytokine or cell population alteration. Future clinical research may focus on stratifying sepsis patients according to specific biomarkers; however, the management of sepsis is a gradual endeavor.

#### IL-3

IL-3 promotes myelopoiesis and cytokine storm in cecal ligation and puncture (CLP)-induced acute sepsis, and inhibiting IL-3 activity protected mice from sepsis-induced increases in neutrophils, inflammatory monocytes, and inflammatory cytokines, reducing organ damage and improving survival [31]. Anti-IL-3 antibody treatment significantly improved survival in septic mice, possibly associated with increased Treg percentage and function [32]. IL-3 has a dual role in sepsis, stimulating innate immune responses that are detrimental in the acute phase but protective in the immunosuppressive phase by improving antiviral defense mechanisms. IL-3 also could protect viral pneumonia in sepsis by promoting the recruitment of circulating plasmacytoid DCs into the lung and T cell initiation [33]. High levels of IL-3 in the plasma of septic patients are associated with increased mortality [31].

#### TLR4

TLR4 deficiency or antibody blockade has been shown to be beneficial and can effectively protect animals from sepsis-induced shock and high mortality [34, 35]. NI-0101 is the first monoclonal antibody to block TLR4 signaling and prevent cytokine release in healthy volunteers after receiving LPS [36]. Patients with high APACHE scores may benefit from eritoran, a synthetic lipodisaccharide that binds to MD2-TLR4 and competitively blocks LPS to TLR4 [37]. Unfortunately, eritoran was withdrawn from further clinical testing in 2011, which failed to be efficacious in clinical trials [38]. In a randomized, double-blind, placebo-controlled trial, TAK-242 treatment did not suppress cytokine levels or reduce 28-day all-cause mortality in patients with severe sepsis [39]. Due to septic patients’ complicated and varied immunological status, TLR4 inhibitors may benefit patients early in the sepsis’ inflammatory phase or in combination with other medicines.

### Glucocorticoids

The first clinical trial using glucocorticoids for sepsis demonstrated a significant mortality reduction in patients with high-dose glucocorticoids [40]. However, later studies reported that short-term administration of high-dose glucocorticoids was associated with worsening secondary infection and increased risk of death, and that low to moderate doses of glucocorticoids also did not improve survival or shock reversal in sepsis [41–44]. Combining hydrocortisone with fludrocortisone significantly reduced 90-day all-cause mortality in patients with septic shock [45]. A recent meta-analysis of metabolic resuscitation with vitamin C, glucocorticoids, vitamin B1, or a combination of these drugs did not significantly reduce long-term mortality (90 days to 1 year) in adults with sepsis or septic shock compared with placebo/usual care [46]. 25-60% of patients with sepsis experience relative adrenal insufficiency (RAI). Glucocorticoid treatment of mice with CLP-induced sepsis was found to be beneficial in RAI mice but detrimental in mice without RAI [47]. In the first phase 3 trial by Annane et al., low doses of hydrocortisone significantly reduced mortality in patients with septic shock and RAI [48]. Genome-wide profiling of peripheral blood leukocytes from septic patients defined two distinct sepsis response signatures (SRS1 and SRS2) [49, 50]. Antcliffe et al. showed that septic patients with the immunocompetent SRS2 endocrine phenotype had significantly higher mortality with corticosteroids [51]. Genome-wide expression profiling using microarray technology and analytics may target a subclass of patients to benefit most from immunotherapy, providing personalized and precision medicine. Thus, further precision medicine approaches based on the RAI and SRS2 endocrine phenotype in patients with sepsis will probably benefit patients from glucocorticoids treatment, which needs further validation in clinical trials.

### Other immunomodulatory drugs

Ulinastatin (UTI) is a multifunctional Kunitz-type serine protease inhibitor with anti-inflammatory and neuroprotective effects [52]. Intravenous administration of UIT inhibits inflammatory mediators and lymphocyte apoptosis levels in CLP-model mice with sepsis [53]. A retrospective study found that the use of UTI in 263 patients with severe sepsis reduced mortality by 23.5% [54]. However, in a multicenter randomized controlled study, UIT treatment reduced all-cause mortality at 28 days in a multivariate analysis, however there was no statistical difference in mortality in the intention-to-treat analysis [55]. A meta-analysis including 13 studies showed that UIT improved all-cause mortality, APACHE II scores, and inflammatory cytokine profiles in patients with sepsis or septic shock [56]. There remains an urgent need for larger randomized clinical trials to evaluate the impact of UTI in patients with sepsis.

Xuebijing (XBJ) injection is a Chinese herbal medicine containing extracts from five herbs, and it has been incorporated into the routine sepsis care in China since 2004. XBJ inhibited inflammation and regulated Tregs/Th17 in various animal models of sepsis [57–59]. Our previous multicenter, randomized, double-blind, placebo-controlled trial showed that treatment with XBJ reduced 28-day mortality in patients with sepsis compared to the placebo group [60]. A meta-analysis including 16 randomized controlled trials demonstrated that XBJ combined with routine treatment improved 28-day mortality in patients with sepsis [61]. In China, approximately 250,000 patients are treated with XBJ each year, and XBJ has been shown to be safe and well tolerated. Although XBJ is a potentially effective treatment for sepsis, additional research is still needed to understand its pharmacokinetics, interactions with antibiotics and pharmacological mechanisms of action.

Macrolides are a class of antimicrobials primarily against Gram-positive cocci and atypical pathogens. However, growing evidence shows that macrolides can be used as modulators of the host immune response in sepsis [62, 63]. Clarithromycin accelerated the resolution of ventilator-associated pneumonia (VAP) and weaning from mechanical ventilation in patients with sepsis and VAP [64]. Compared to the placebo group, sepsis patients in the clarithromycin group showed a decrease in serum IL-10 to TNF-α ratio and restoration of the balance between pro- and anti-inflammatory mediators [65]. Intravenous clarithromycin did not affect overall mortality in patients with sepsis, but clarithromycin may provide long-term survival benefits while also reducing the cost of hospitalization for patients [66, 67].

### Enhance immunity

#### Immunostimulatory factor

##### G-CSF and GM-CSF

GM-CSF has been shown to reverse monocyte hyporesponsiveness in vitro and in vivo studies to increase blood monocyte levels, upregulate monocyte responsiveness, and increase HLA-DR expression, which is known to enhance antigen presentation and adaptive immune responses [68–70]. Premature neonates with sepsis or/and neutropenia treated with rhG-CSF adjuvant therapy were discharged with lower all-cause mortality and faster recovery of total leukocytes and ANC [71, 72]. Marlow et al. administered subcutaneous GM-CSF at a dose of 10 μg/kg daily for five days to infants less than 31 weeks of gestation and small-for-gestational-age (SGA), and showed no adverse outcomes during subsequent 2- and 5-year follow-up periods [73, 74]. However, a meta-analysis showed that a significant increase in the reversal rate of infection with G-CSF or GM-CSF therapy, patients patients with severe sepsis/septic shock did not have a benefit in 14- or 28-day mortality [75]. Meisel et al. treated patients with sepsis (monocytic HLA-DR [mHLA-DR] <8,000 monoclonal antibodies (mAb) per cell for 2 d) GM-CSF and observed a trend toward improved disease severity and restoration of mHLA-DR expression and cytokine release [76]. G-CSF or GM-CSF application may lead to different outcomes in different stages of severe sepsis and is more applicable in patients with severe immunosuppression, making individualized and precise therapy guided by biomarkers based on immune status extremely important.

#### Tα1

Thymosin alpha 1 (Tα1) is an endogenous modulator of the innate and adaptive immune system. Tα1 plays an important biological role in activating and restoring sepsis in patients with a dysregulated immune response [77]. Tα1 may effectively improve the prognosis of patients with severe sepsis, improving HLA-DR expression, and reducing the incidence of secondary infections [78, 79]. In a meta-analysis of whether Tα1 was used in combination with UTI, the combination of UTI and Tα1 for severe sepsis reduced mortality at 28 and 90 days, whereas Tα1 alone reduced mortality only at 28 days [80]. Tα1 may be more effective as an immune modulator in patients with immunosuppressed states. A recent clinical trial (NCT02867267) in China further evaluated the efficacy and safety of Tα1 for sepsis, and recruitment in the study is now complete, and some information can be brought to light through this study.

#### IFN-γ

IFN-γ may be harmful during the pro-inflammatory phase of sepsis, which can stimulate monocytes and cause a vicious cycle of hyperinflammation [81]. However, IFN-exogenously administered would reverse markers of monocyte deactivation and ameliorate post-sepsis immunosuppression. 18 healthy male volunteers were treated with IFN-γ or GM-CSF or placebo after intravenous administration of Escherichia coli endotoxin, and IFN-γ partially reversed human immunoparalysis [82]. Patients with invasive Candida and/or Aspergillus infections regained partial immune function after IFN -γ treatment [83]. Classifying sepsis patients into independent immune classification strata based on ferritin and HLA-DR receptors/monocytes may improve the chances of successful immunotherapy trials in sepsis [84]. Patients with increased monocyte HLA-DR expression after IFN-γ treatment early (<4 days) or late (>7 days) after a sepsis episode improved immune host defense in sepsis-induced immunosuppression [85]. IFN-γ may be a potential immunomodulatory therapy to reverse immunoparalysis in vivo in humans during sepsis.

#### IL-7

In several models of sepsis infection involving bacteria, fungi, and viruses, IL-7 treatment blocked CD4 and CD8T cell apoptosis, restored IFN- and immune effector cell recruitment, and improved mouse survival [86–88]. IL-7 levels are decreased in patients with sepsis [89]. IL-7 immunotherapy improved clinical symptoms, cleared the fungus, reversed lymphopenia, and reversed the profound loss of CD4+ and CD8+ T cells induced by sepsis [90–92]. Bidar et al. found that patients with severe COVID-19 admitted to the ICU exhibited severe T-cell depletion, which could be reversed in vitro by rhIL-7 [93]. In some cases, reports showed that IL-7 could be safely used in patients with severe COVID-19 and absolute lymphocytopenia and can benefit patients [94, 95]. IL-7 is safe and well tolerated and is a promising new immune-adjuvant therapy for sepsis.

#### IL-15

IL-15-deficient (IL-15 KO) mice are resistant to septic shock but IL-15 treatment exacerbates the severity of sepsis by activating NK cells and promoting IFN-γ production. Masafumi et al. showed that three subcutaneous injections of 1.5 μg IL-15 enhanced long-term T-cell depletion, increased NK and macrophage levels, and reduced mortality in mice [96]. The levels of plasma IL-15 were modestly increased and increased mortality in patients with severe lymphopenia compared to patients without lymphopenia [97]. Elevated serum IL-15 levels in patients with sepsis after emergency abdominal surgery were associated with prognosis and organ dysfunction, with non-survivors having significantly higher basal IL-15 levels than survivors, and this difference persisted throughout the course of the study [98]. Although IL-15 has a stimulatory effect on many immune cells, it may also promote systemic inflammation and organ damage in treating sepsis and has also been shown to have potentially toxic effects. Therefore, IL-15 needs further study as an immunotherapeutic agent in sepsis.

#### IL-33

IL-33 treatment played a protective activity against sepsis and also significantly reduced mortality in CLP septic mice [99–101]. IL-33 promotes inflammation by binding to its receptor ST2 (IL1RL1), expressed primarily on immune cells, making the IL-33/ST2 axis a bridge between immune system coordination and tissue damage [102]. Administration of IL-33Rα (ST2)-blocking antibody reduced IL-10 levels 24 hours after CLP, and a survival benefit was observed within 72 hours [103]. ST2 deletion affected septic dendritic cells’ phenotype and maturation and downregulated myeloid precursors and inflammatory NK cells [104]. During the immunosuppressive phase of sepsis, IL-33 levels increased and remained high for five months after recovery [105]. IL-33/ST2 is a novel axis associated with poor immune function in sepsis, and this axis may benefit patients as a personalized treatment for sepsis.

### Immune checkpoint

#### PD-1/PD-L1

Upregulation of programmed cell death protein 1 (PD-1) on neutrophils may be associated with sepsis-induced immunosuppression [106]. Huang et al. have demonstrated that the survival of PD-1−/− mice is improved in a mouse model of sepsis induced by the cecal ligation-and-puncture procedure [107]. By blocking PD-L1 in animal models of sepsis, lymphocyte apoptosis was inhibited, macrophage dysfunction was reversed, and survival was improved [108, 109]. Treatment with anti-PD-1 and anti-PD-L1 specific antibodies prevented and/or reversed T-cell depletion and reversed immune dysfunction [110]. Our previous study showed that PD-1 expression on memory CD8+ T cells identifies patients with a poor prognosis during sepsis [111]. In the first clinical evaluation in sepsis, the anti-PD-L1 immune checkpoint inhibitor appeared to be well tolerated and had the potential to restore immune status [112]. Immunotherapy has been shown to be effective in clinical trials for several types of malignancies, with FDA-approved anti-PD-1 blocking antibodies like nivolumab and pembrolizumab [113]. Nivolumab therapy also appeared to be well tolerated and safe in treating patients with sepsis or septic shock [114, 115]. Using nivolumab appears to improve selected immune markers such as ALC and monocyte human leukocyte antigen-DR subtype transcript levels. Immune checkpoints play an important role in sepsis, but immune checkpoint modulation strategies for sepsis still need to be further refined and personalized to balance the immune status of patients to prevent immune disorders.

#### CTLA-4

Cytotoxic T-lymphocyte-associated protein 4 (CTLA-4) is a coinhibitory cell surface protein expressed on T cells. Thus, overexpression of CTLA-4 downregulates T cell activation and proliferation and suppresses the host immune response, preventing an overreaction of the immune system. Inoue et al. showed increased expression of CTLA-4 on CD4, CD8, and regulatory T cells in a CLP mouse model, resulting in significantly improved survival at low doses and worsened survival at high doses when anti-CTLA-4 treatment was administered [116]. Chang et al. confirmed that immuno-adjuvant therapy with anti-PD-1, anti-PD-L1 and anti-CTLA-4 antibodies reversed sepsis-induced immunosuppression and improved survival in mice [117]. CTLA-4 is also highly expressed in CD4 T cells, CD8 T cells, and/or inhibitory receptors in monocytes from patients with sepsis [118, 119]. CTLA-4 genetic variants can be an important predictor of survival in sepsis patients, and precise anti-CTLA-4 therapy can be stratified according to CTLA-4 gene variants [120, 121].

#### TIM-3

T cell immunoglobulin and mucin domain-containing protein 3 (TIM-3) have been suggested to play an important role in maintaining immune homeostasis in sepsis. TIM-3 gene variants were associated with altered 28-day mortality and susceptibility to Gram-positive infections in patients with sepsis [122]. Upregulation of Tim-3 expression is associated with poorer disease severity and prognosis in sepsis patients [123]. Blocking the Tim-3/Galectin-9 signalling axis inhibits NKT apoptosis in sepsis [123]. Huang et al. found that Tim-3 regulates sepsis-induced immunosuppression by inhibiting the NF-κB signalling pathway in CD4 T cells [124]. Blocking the immune checkpoint molecule Tim-3 may be a promising immunomodulatory strategy for the future clinical treatment of sepsis.

#### LAG-3

Lymphocyte activation gene 3 (LAG-3) is an immunoregulatory cell surface protein that negatively regulates T cell proliferation, activation, and homeostasis and is highly expressed in sepsis [118]. Genetic variation in LAG-3 is associated with altered disease severity and outcome in patients with sepsis, and 28-day mortality is significantly lower in LAG-3 rs951818 AA-homozygote patients than in C allele carriers [125]. LAG-3 knockout or anti–LAG-3 antibody blockade protected mice undergoing CLP from sepsis-associated immune dysfunction and maybe a new target for the treatment [126].

#### TIGIT

TIGIT is a novel coinhibitory molecule expressed on peripheral memory and regulatory CD4+T cells and NK cells. TIGIT was upregulated in Treg and NK cells of healthy and cancer sepsis mice [127]. Furthermore, the anti-TIGIT antibody reversed sepsis-induced T cell apoptosis in cancer septic mice and increased their 7-day survival in cancer septic mice. Expression of TIGIT on T cells was significantly upregulated in sepsis patients, and in vitro blockade of TIGIT using an anti-TIGIT antibody restored the frequency of cytokine-producing T cells in sepsis patients [128]. Our previous study showed that the anti-TIGIT Ab reversed sepsis-induced T-cell apoptosis in cancer septic mice and resulted in a significant survival benefit [129].

#### VISTA

V-domain Ig suppressor of T cell activation (VISTA) has been identified as an immune checkpoint molecule that negatively regulates T-cell activation. Treatment with a high-affinity anti-VISTA antibody (clone MH5A) improved survival in septic mice and resulted in reduced lymphocyte apoptosis, decreased cytokine expression, and increased bacterial clearance [130]. Gray et al. showed that VISTA could regulate CD4+ Treg in response to an infectious attack during sepsis progression, exerting a protective effect and reducing septic morbidity/mortality [131]. VISTA also induces tolerance and transcriptional reprogramming of the anti-inflammatory program in macrophages to attenuate innate inflammation in vivo [132].

### Immunoglobulin

Low levels of immunoglobulins are positively associated with the severity of critical illness and mortality in patients with sepsis [133]. A recent meta-analysis showed that the use of intravenous IgM-enriched immunoglobulins (IVIgGM) in adult sepsis patients may be associated with reduced mortality and shorter mechanical ventilation lengths [134]. However, in 2021, the Surviving Sepsis Campaign guidelines recommend against intravenous immunoglobulins in patients with sepsis or septic shock due to low quality of evidence [135]. Martinez et al. showed in a case-control analysis that treatment with IgGM in patients with sepsis significantly reduced 28-day mortality based on a validated selection of severity-matched comparators [136]. Patients with sepsis with low IgG levels (<670 mg/dL) had significantly lower mortality with intravenous immunoglobulin at 28 and 90 days [133]. Early administration (within 12 hours) of IgM- and IgA-enriched intravenous polyclonal immunoglobulins reduced the risk of in-ICU mortality in patients with septic shock caused by any pathogens [137]. There is a pressing need for more precise use of immunoglobulins in terms of patients’ selection, dosage, and timing in sepsis, which has to be verified by future clinical studies and may provide the greatest benefit.

### Mesenchymal stem cell

Mesenchymal stem cells (MSCs) have attracted attention for sepsis treatment due to their anti-inflammatory and tissue regenerative potential to modulate innate and adaptive immune systems. MSCs can also act distantly on their targets through paracrine and extracellular vesicles (EVs) secretion-mediated pathways. Allogeneic adipose-derived MSCs (ADSCs) in CLP model mice at a dose of 2 × 107 cells/kg significantly reduced mortality, bacterial load, systemic inflammation, and multi-organ damage [138]. MSC-derived EVs attenuated pulmonary edema and inflammation in acute lung injury induced by an LPS-induced sepsis model in mice [139]. The use of umbilical cord-derived human MSCs for treating 15 patients with severe sepsis was well tolerated in the first phase 1 clinical trial [140]. Perlee et al. infused healthy subjects with allogeneic adipose MSCs (ASCs) followed by intravenous (2 ng/kg) LPS and found that high doses of MSCs were able to increase pro- and anti-inflammatory factors during the process [141]. Infusion of MSCs in patients with septic shock did not elevate cytokine levels and organ damage while dose-dependently attenuating pro-inflammatory cytokines [142]. MSCs may be a safe and effective strategy to treat sepsis, however, large-scale randomized controlled studies are required to convince present evidence.

### Discussion and future perspectives

Sepsis is a dynamic disorder of dysregulated inflammatory and immune responses. Our previous study employed machine learning and bioinformatics to evaluate genome-wide gene expression profiles in sepsis patients’ blood to construct a model that efficiently classifies sepsis into immunoparalysis and immunocompetent endotypes [143]. Seymour et al. sorted sepsis into four clinical phenotypes after a retrospective analysis of all clinical and laboratory variables in the electronic health records of 20,189 patients with sepsis using machine learning [144]. Baghela et al. used machine learning and data mining to analyze gene expression signatures to classify patients with early sepsis into five distinct mechanistic endotypes [145]. However, there is still a large gap between the creation of AI algorithms and clinical implementation, and further prospective studies in multiple clinical settings are needed to improve the generalizability of these AI models.

The application of immune monitoring in treating patients with sepsis may facilitate early identification and diagnosis, allowing pre-emptive action to reduce the risk of secondary infection, organ dysfunction, and death. Using biomarkers to personalize and monitor therapy allows physicians to modulate the immunity of sepsis patients in real-time with selective immunotherapeutic agents. The following points may be noted for the use of immunotherapeutic agents in sepsis. Firstly, we need to be aware that unnecessary suppression of immune checkpoints can disrupt normal immune homeostasis and may cause side effects such as inflammation and autoimmune diseases. Therefore, the mechanisms of action of therapeutic agents and their adverse effects need to be precisely elucidated. Secondly, phenotypic analysis of the patient’s immune status and the development of a panel of biomarkers to enable targeted immunomodulatory interventions against sepsis-induced immune alterations. Finally, establishing rapid bedside testing, where targeted therapies will progress with the different stages of sepsis rather than being limited to treatments based on clinical presentation, makes sepsis treatment a prospective strategy.

## Conclusion

Clinical trials have demonstrated that the use of “one-target” and “one-size-fits-all” treatment plans is unlikely to be effective for sepsis due to the intricate host response and the diverse pathophysiological changes. Therefore, it is important to develop an early warning system that can help us better understand the intricate biology, genetics, immunology, and clinical factors involved in sepsis to achieve accurate clinical phenotypes and precision treatment. Subsequent research endeavors should aim to enhance the comprehension of sepsis immune status, monitor immune progression, identify biochemical and immune risk factors, and explore biomarkers in sepsis patients. Furthermore, clarifying clinical and immune stratification and implementing strategies for AI-assisted clinical translation are essential for advancing sepsis management.

### Supplementary information

Supplemental Table 1

## Data Availability

There are no experimental datasets given that this is a review article that is prepared based on a literature review.
