# Supplementary material for: Immune dysregulation in sepsis: experiences, lessons and perspectives
Source: Cell Death Discov. 2023 Dec 19;9:465. doi: 10.1038/s41420-023-01766-7 (PMC10730904; doi:10.1038/s41420-023-01766-7)
Supplement: Supplementary file 1 — Supplemental Table 1 [file 41420_2023_1766_MOESM1_ESM.docx]

**Supplemental Table 1. Clinical studies that aim to modulate immune response and enhance immunity in sepsis. (2010-2023)**

| **Treat-ment** | **Mechanism of action** | **Study design** | **Clinical trials** | **Sample Size** | **Primary outcome/objectives** | | **Author conclusion** | **Potential adverse effects** | **Potential Benefit Population** |
| --- | --- | --- | --- | --- | --- | --- | --- | --- | --- |
| **Modulate immune response** | | | | | | | | | |
| Anakinra | Blocks IL-1 | A phase III, randomized, double-blind, placebo-controlled, multicenter trial (reanalysis) | N[1] | 763(HBD/DIC=43; Non- HBD/DIC=720) | 28-day survival | IL-1 receptor blockade was associated with significant improved survival in patients with sepsis and concurrent hepatobiliary dysfunction/disseminated intravascular coagulation. | | Non-serious adverse reactions | Macrophage activation syndrome |
|  |  | A double-blind, double-dummy randomized clinical study | NCT03332225[2] | 36 (placebo=21; immunotherapy=15) | 28-day survival | 42.9% of patients survived after 7 days with SOFA score decrease (p = 0.042). Anakinra treatment was promising in personalized immunotherapy. | | There was no difference in the incidence of adverse events between the two arms of the study. | Macrophage activation-like syndrome and immunoparalysis |
| TNF alpha MAb | Blocks pro-inflammatory effects of TNFa | A placebo-controlled, double-blind, dose-escalation study | NCT00615017[3] | 70 (AZD9773 = 47; placebo = 23) | Determine the safety and tolerability of AZD9773, anti-human TNF-α Fab preparation, in patients with severe sepsis. | AZD9773 was well tolerated. | | Most mild-to-moderate adverse events were considered by the investigators unrelated to study therapy. | N |
|  |  | A phase II, double-blind, placebo-controlled, dose-escalation study | NCT01144624[4] | 20 (AZD9773 250/50 U/kg, n = 7; AZD9773 500/100 U/kg, n = 7; placebo, n = 6 | Safety/tolerability and pharmacokinetics | Treatment with AZD9773 led to a decrease in TNF-α concentrations. AZD9773 was generally well tolerated with dose-proportional pharmacokinetics in Japanese patients with severe sepsis/septic shock. | | All patients experienced at least one TEAE  ; the most common TEAEs in the  AZD9773 cohorts were pleural effusion  and peripheral edema; acute myocardial infarction, ventricular tachycardia, cerebral infarction, parkinsonism, large  intestinal perforation, and gastric cancer. | N |
|  |  | A randomized, double-blind, placebo-controlled phase IIb study | NCT01145560[5] | 296(AZD9773(low-dose=100; high-dose=97; placebo=99) | Mean number of ventilator-free days. evaluate the effect of AZD9773 at low and high doses versus placebo on the number of ventilator-free days over 28 days | AZD9773 rapidly and effectively reduces circulating TNF-α in patients with severe sepsis/septic shock, but this remarkable pharmacodynamic effect does not appear to lower proinflammatory cytokines (IL-6 and IL-8), nor does it appear to have a clinical benefit. | | Most patients experienced at least one TEAE during the study although the majority of TEAEs were mild or moderate in nature. erythema and generalized erythema. | N |
| TLR4 antagonists | Blocks TLR4 | A Prospective, randomized, double-blind, placebo-controlled, multicenter, ascending-dose phase II trial | N[6] | 235(low-dose=80; high-dose=77; placebo=78) | The 28-day all-cause mortality rate in the MITT group | Treatment with eritoran tetrasodium seemed to be well tolerated. High doses of eritoran tetrasodium tended to lower mortality in patients with severe sepsis and a high APACHE II score. | | Anemia, diarrhea, insomnia, acute renal failure, and rash | N |
|  |  | A randomized, double-blind, placebo-controlled trial. | N[7] | 223(low-dose=74; high-dose=75; placebo=74) | 28-day all-cause mortality rate; change in serum interleukin-6 levels relative to baseline | TAK-242 did not suppress cytokine levels in sepsis, shock, or respiratory failure patients. | | Anemia, methemoglobinemia, hypokalemia, pyrexia, and urinary tract infections | N |
|  |  | A randomized, double-blind, placebo-controlled, multinational phase 3 trial | NCT00334828[8] | 1962(eritoran=1305; placebo=657) | 28-day all-cause mortality | Eritrean did not decrease mortality at 28 days in patients with severe sepsis compared to placebo. | | There was no significant difference in the overall incidence of adverse events. | N |
| Glucocorticoids | Immunosuppressive and reduce levels of pro-inflammatory mediators | A retrospective cohort study | ISRCTN99675218[9] | 75(hydrocortisone=39; placebo=36) | 28-day all-cause mortality | The trial was terminated for futility at interim analysis. Hydrocortisone treatment initially had a positive effect on hemodynamic parameters, but did not reduce mortality and was associated with an increase in adverse effects. | | Gastrointestinal bleeding | N |
|  |  | A Prospective, randomized, double-blind study | N[10] | 130(3-day treatment=65; 7-day treatment=65) | 28-day mortality | There was no difference in 28-day mortality between 3 or 7 days of low-dose hydrocortisone treatment in patients with septic shock and RAI. | | There was no significant difference in the overall incidence of adverse events. | N |
|  |  | A prospective open-label randomized controlled pilot trial. | ISRCTN66727957[11] | 61(vasopressin + hydrocortisone=31; vasopressin + placebo=30) | The difference in plasma vasopressin concentration between treatment groups. | Hydrocortisone reduced vasopressin requirements and reduced duration in the treatment of sseptic shock, but did not alter plasma vasopressin levels. | | Extension of a preexisting recent ischemic cerebral infarct, cool/mottled peripheries, and an increase in serum lactate and troponin. | N |
|  |  | A factorial (2×2), double-blind, randomized clinical trial | ISRCTN 20769191[12] | 409(vasopressin and hydrocortisone =101; vasopressin and placebo=104; norepinephrine and hydrocortisone=101; norepinephrine and placebo=103) | kidney failure-free days during the 28-day period after randomization | There was no difference in mortality or serious adverse events between the hydrocortisone and placebo groups. | | N | N |
|  |  | A double-blind, single-center, randomized, placebo-controlled trial | NCT01284452[13] | 197(hydrocortisone=98; placebo=99) | 28-day all-cause mortality | In sepsis-associated ARDS, hydrocortisone treatment was associated with improved lung function, but there was no significant survival benefit for patients. | | Hyperglycemia | N |
|  |  | A multicenter, prospective, randomized, double-blind, pilot study | NCT02768740[14] | 122(200-mg group = 59; 300-mg group = 63) | 28-day mortality | In patients with septic shock, there was no significant difference in mortality or adverse events between the two distinct hydrocortisone dosing regimens, but it was associated with infection persistence and the use of etomidate. | | There was no significant difference in the overall incidence of adverse events. | N |
|  |  | A double-blind, randomized clinical trial | NCT00670254[15] | 380(hydrocortisone=190; placebo=190) | Development of septic shock within 14 days | Hydrocortisone did not reduce the risk of septic shock within 14 days compared with placebo in patients with severe sepsis who did not experience septic shock. | | Muscle weakness, weaning failure, secondary infection, and gastrointestinal bleeding. | N |
|  |  | A randomized, double-blind, placebo controlled trial | NCT02044159[16] | 49(hydrocortisone=23; placebo=26) | Determine the rate of patient accrual | It may be feasible to conduct a large randomized controlled trial to assess the effectiveness of using corticosteroids early in pediatric septic shock. | | There was no significant difference in the overall incidence of adverse events. | N |
|  |  | A double-blinded randomized controlled trial | NCT02266264[17] | 80(100-mg group = 40; 200-mg group = 40) | Compare the occurrence of hyperglycemia after hydrocortisone initiation (defined as peak blood glucose greater than or equal to 180 mg/dL) | Patients with septic shock treated with 100 mg of hydrocortisone can reduce hyperglycemia and steroid-related adverse effects without increasing mortality compared with 200 mg. | | Hyperglycemia, glycemic variability and immunosuppression. | N |
|  |  | An investigator-initiated, international, pragmatic, double-blind, parallel-group, randomized, controlled trial | NCT01448109[18] | 3658(hydrocortisone=1832; placebo=1826) | 90-day all-cause mortality | Continuous infusion of hydrocortisone did not reduce 90-day mortality in patients with septic shock undergoing mechanical ventilation. | | Hyperglycemia, hypernatremia, hyperchloremia, hypertension, bleeding, encephalopathy, leukocytosis, myopathy and septic arthritis. | N |
|  |  | A multicenter, double-blind, randomized trial | NCT00625209[19] | 1241(hydrocortisone-plus-fludrocortisone=614; placebo=627) | 90-day all-cause mortality | Lower 90-day all-cause mortality in patients with septic shock treated with hydrocortisone plus fludrocortisone than in placebo-treated patients. | | There was no significant difference in the overall incidence of adverse events. | N |
|  |  | A randomized controlled, open-label trial. | N[20] | 58(hydrocortisone 200 mg/d by continuous infusion=29; 50 mg every 6 h throughout the prescription of vasopressors=29) | shock reversal on day 7 | On day 7, intermittent bolus administration of hydrocortisone was associated with greater shock reversal than continuous infusion. | | There was no significant difference in the overall incidence of adverse events. | N |
|  |  | A retrospective cohort study | N[21] | 287(hydrocortisone=98; placebo=189) | 28-day mortality | In neutropenic patients with septic shock, hydrocortisone was not related to a reduction in 28-day mortality. | | There was no significant difference in the overall incidence of adverse events. | N |
|  |  | A multicenter, open-label, randomized clinical trial | NCT03333278[22] | 216(vitamin C, hydrocortisone, and thiamine=109; hydrocortisone =107) | Duration of time alive and free of vasopressor administration up to day 7 | Intravenous vitamin C, hydrocortisone, and thiamine did not significantly improve survival time in patients with septic shock compared with intravenous hydrocortisone alone. | | No serious adverse events were reported. | N |
|  |  | A randomized, double-blinded, placebo-controlled trial | NCT03422159[23] | 137287(hydrocortisone, ascorbic acid, thiamine=68; placebo=69) | Resolution of shock and change in SOFA score | Intravenous ascorbic acid, thiamine, and hydrocortisone resulted in faster reversal of shock in patients with septic shock compared to intravenous hydrocortisone alone | | No adverse  events were noted that were deemed related to the study  drug. | N |
|  |  | A randomized, blinded, multicenter clinical trial | NCT03389555[24] | 205(ascorbic acid, corticosteroids, and thiaminee=103; placebo=102) | change in the SOFA score (range, 0-24; 0 = best) between enrollment and 72 hours | Ascorbic acid, corticosteroids, and thiamine did not reduce SOFA score in septic shock patients compared to placebo in the first 72 hours after enrolment. | | Hyperglycemia, hypernatremia, and new hospital-acquired infection | N |
|  |  | A multicenter, randomized, double-blind, adaptive-sample-size, placebo-controlled trial | NCT03509350[25] | 501(vitamin C, hydrocortisone, and thiamine=252; placebo =249) | The number of consecutive ventilator- and vasopressor-free days in the first 30 days following the day of randomization | Vitamin C, thiamine, and hydrocortisone did not substantially increase ventilator- and vasopressor-free days within 30 days in critically sick sepsis patients compared to placebo, and the trial was terminated early. | | Hemorrhagic shock and worsening kidney function | N |
|  |  | A prospective, randomised clinical study | NCT04508946[26] | 94(hydrocortisone, thiamine and vitamin C=47; hydrocortisone =249) | 28 day in-hospital mortality and ICU mortality. | Compared to hydrocortisone alone, vitamin C, thiamine, and hydrocortisone did not reduce 28-day mortality in septic shock. | | N | N |
|  |  | A single-center, double-blind, randomized controlled clinical trial | NCT03872011[27] | 408(hydrocortisone, thiamine and vitamin C=47; hydrocortisone =249) | 90-day mortality | Early hydrocortisone, vitamin C, and thiamine combination therapy did not improve survival in adult septic shock patients compared to placebo. | | Hypernatremia, fluid overload, and blood glucose disturbance | N |
| Polymyxin B | Immunomodulation | A prospective, open-labeled, multicenter cohort study | N[28] | 92(early group (PMX within 6 h) =41; late group(after 6 h)=51) | 28-day mortality | Early administration of PMX reduced the duration of ventilator support and the need for catecholamines. | | N | N |
|  |  | A prospective, multicenter, randomized controlled trial | NCT01222663[29] | 232(PMX HP=119; conventional=113) | 28-day mortality | There was a non-significant increase in mortality and no improvement in organ failure with PMX HP treatment of peritonitis-induced septic shock compared to conventional treatment. | | Hemorrhagic episodes | N |
|  |  | A retrospective study | UMIN000012748[30] | 413(PMX HP=134; conventional=279) | 28-day mortality | PMXHP did not reduce 28-day mortality in patients with septic shock caused by Gram-negative bacilli. | | N | N |
|  |  | A retrospective study | N[31] | 143(PMX HP=103; conventional=30) | Assess the safety and effectiveness of selective endotoxin adsorption | PMXHP treatment improved 28-day survival in patients with sepsis. | | No adverse  events were noted in the study. | N |
|  |  | A multicenter, randomized clinical trial | NCT01046669[32] | 450(PMX HP=224; sham hemoperfusion plus standard therapy=226) | 28-day mortality | In patients with septic shock and high endotoxin activity, polymyxin B hemoperfusion treatment plus conventional medical therapy did not reduce mortality at 28 days compared to sham treatment plus conventional medical therapy. | | Deep venous thrombosis; venous air embolism | N |
|  |  | A randomized controlled trial | NCT02413541[33] | 59(PMX HP=29; non-PMX-HP=30) | The change in mHLA-DR expression | The expression of mHLA-DR was increased by PMX-HP in patients with severe sepsis. | | No adverse events have been attributed to this intervention. | Low mHLA-DR expression |
|  |  | A prospective nationwide cohort | Osaka General Medical Center IRB No. 30-S11-004[34] | 741(PMX HP=92; non-PMX-HP=625) | all-cause in-hospital mortality | Even though PMX-HP did not lower in-hospital mortality for all septic shock patients, it might help a small group of people with advanced age and more severe disease. | | N | High age and higher disease severity |
| Ulinastatin | A serine protease inhibitor, inhibits several pro-inflammatory proteases | A multicenter randomized controlled study | CTRI/2009/091/000650[35] | 114(ulinastatin=55; placebo=59) | 28-day all-cause mortality | In the modified intention-to-treat analysis, intravenous administration of ulinastatin reduced mortality in patients with severe sepsis, but not in the intention-to-treat analysis. | | Intracranial hemorrhage | N |
|  |  | A prospective, randomized, single-blind trial | N[36] | 75(UIT=19, Rhubarb=19,UIT+ Rhubarb=21,placebo=16) | Investigate the effect of the combination treatment of UTI and rhubarb | All treatments (except the control group) significantly lowered C-reactive protein, leukocyte density, lactate, and APACH II scores and boosted CD4/CD8 levels. UTI plus rhubarb also dramatically reduced calcitoninogen levels. | | N | N |
| Xuebijing | Immunomodulation | A randomized, double-blind, and placebo-controlled trial | N[37] | 69(Xuebijing =34, placebo =35) | Investigate the protective effect and possible mechanism of Xuebijing Injection on myocardial injury in patients with sepsis, and evaluate its prognostic implications. | Xuebijing injection protected septic myocardial injury and increased cardiac troponin I, N-terminal proB-type natriuretic peptide, and procalcitonin levels. | | No adverse events have been attributed to this intervention. | N |
|  |  | A case-control study | N[38] | 100(CVVH=50,CVVH+ Xuebijing=50) | Evaluation of Curative Effect and Mortality. | The mortality rate of CVVH+Xuebijing was lower than that of the control group, and the treatment of severe sepsis had positive significance. | | N | N |
|  |  | A multicenter, randomized double-blind, placebo-controlled trial | NCT03238742[39] | 1817(Xuebijing =911, placebo =906) | 28-day mortality | Compared to the placebo, the administration of XBJ resulted in a lower 28-day death rate. | | There was no significant difference in the overall incidence of adverse events | N |
| Clarithromycin | Immunomodulation | A double-blind, randomized, multicentre trial | NCT01223690[40] | 600(clarithromycin=302; placebo=298) | 28-day mortality | The administration of intravenous clarithromycin had no effect on overall mortality; however, it shortened the time to infection resolution and decreased hospital costs. | | Allergic rash, pain at the infusion site and of superficial thrombophlebitis | N |
|  |  | A prospective, double-blind, randomized, placebo-controlled, multicenter clinical trial | NCT00297674[41] | 200(clarithromycin=100; placebo=100) | 90-day mortality | As an adjunctive treatment for VAP and sepsis, intravenous clarithromycin for three consecutive days offers long-term survival benefits and a substantial reduction in hospitalization costs. | | No adverse events have been attributed to this intervention. | N |
|  |  | A multicenter, randomized, clinical trial | NCT03345992[42] | 110(clarithromycin=55; placebo=55) | 28-day all-cause mortality | Clarithromycin did not decrease mortality in sepsis patients with respiratory and multiple organ dysfunctions. However, clarithromycin was associated with a decreased risk of sepsis recurrence, presumably through an immune-restoration mechanism. | | There was no significant difference in the overall incidence of adverse events. | N |
| **Enhance immunity** | | | | | | | | | |
| G-CSF | Increased total leukocyte counts; stimulates the production of neutrophils and modulates the function and activity of developing and mature neutrophils | A Randomized, Single-Blind, Non-placebo-controlled Trial | N[43] | 40(20/group) | Mortality rate | Preterm neonates with sepsis and neutropenia treated with rhG-CSF adjunctive reduced all-cause mortality and accelerates total leucocyte and ANC recovery at hospital discharge. | | N | Preterm neonates with sepsis and neutropenia |
|  |  | A Randomized Controlled Trial | NCT01479114[44] | 60(30/group) | Duration of antibiotic therapy, ventilation,  and hospitalization | Compared to the non-G-CSF group, the G-CSF group had shorter antibiotic and hospitalization durations. G-CSF adjuvant therapy for neonatal septicemia, whether neutropenic or not, improved neutrophil numbers and function and speeded up sepsis recovery. | | All neonates tolerated  rhG-CSF well and no adverse reactions were identified. | N |
|  |  | A randomized case-controlled study | N[45] | 56(rhG-CSF+ antibiotic =33; antibiotic=23) | Sepsis related  short-term mortality | Treatment with rhG-CSF resulted in a more rapid recovery of ANC in neutropenic preterm infants. | | No drug-related adverse event was recorded. | Neutropenic preterm infants |
| GM-CSF | Stimulates the accelerated production of neutrophils, monocytes and macrophages. Induces monocytic cytokine expression and antigen presentation. | A randomised trial | ISRCTN42553489[46, 47] | 280(GM-CSF=139; No GM-CSF=141) | Whether the administration of GM-CSF in the neonatal period produced differences in survival free of severe disability at 2 years of age. General intelligence as measured using the mental processing composite (MPC; equivalent to IQ) of the Kaufmann-ABC (Pearson UK) at 5 years | Prophylactic administration of GM-CSF did not improve neurodevelopment or health at two years, nor did it influence neurodevelopment, general health, or educational outcomes at five years in very preterm SGA babies. | | N | N |
|  |  | An open-label randomized trial | N[48] | 14(7/group) | Immunoparalysis (TNFα <200 pg/mL beyond day 3 of MODS) can be reversed with GM-CSF. | GM-CSF therapy facilitated rapid recovery of TNFα response to >200 pg/mL by 7 days and prevented nosocomial infection. | | No GM-CSF-related adverse events were observed. | N |
|  |  | A Double-Blind, Placebo-controlled, Randomized Pilot Study | NCT 01374711[49] | 18(Placebo=6; GM-CSF=6; IFN-γ= 6) | To investigate the effects of IFN-γ and GM-CSF on immunoparalysis in vivo in humans. | IFN-γ partially cured immunoparalysis in vivo, and the effects of GM-CSF tended to be similar to those of IFN-γ. | | No serious adverse events occurred during the trial. | N |
| Thymosin alpha 1 | Stimulates endogenous IFN-γ secretion, and enhances T cells and the whole immune system | A multicenter, single-blind, randomized and controlled trial | NCT00711620[50] | 361 (control = 180; Tα1 = 181)) | 28-day mortality | Thymosin alpha 1 therapy with standard medication therapy may improve clinical outcomes in severe sepsis patients. | | No serious drug-related adverse event was recorded. | N |
|  |  | A randomized controlled study | N[51] | 86(43group) | Preparation  time before treatment, symptom-onset-to-door (SOTD),  duration of shock, length of stay in the intensive care unit  (ICU), and incidence of adverse reactions. | T-α1 and blood purification improved immunological, myocardial, and inflammatory function in septic shock patients. | | There was no significant difference in the overall incidence of adverse events. | N |
| IFN-γ | Increased mHLA-DR expression in monocytes, promoted secretion of inflammatory cytokines, attenuated production of the IL-10. | An open-label, prospective case series | NCT01270490[52] | 6(3/group) | The effects of treatment with rIFN-γ on the host innate and adaptive immune responses. | Patients with invasive Candida and/or Aspergillus infections regained partial immune function after IFN -γ treatment. | | No other significant adverse events were observed. | IFN-γ |
|  |  | An open-label prospective multi-center case-series | NCT 01649921[53] | 20 | The effects of interferon gamma therapy in sepsis induced immunosuppression | IFN-γ immunotherapy appeard to be well tolerated and increased immunological host defense in sepsis-induced immunosuppression. | | No serious adverse events linked to IFNγ treatment were observed. |  |
| IL-7 | Improve lymphocyte functionality and increased CD4^+^ and CD8^+^ T cells. | A prospective, randomized, double-blind, placebo-controlled trial | NCT02640807 and NCT02797431[54] | 27(placebo=10; low frequency CYT107=8; high frequency CYT107=8) | Assess the safety and tolerability of recombinant human IL-7 (CYT107) | CYT107 corrected sepsis patients' severe CD4+ and CD8+ deficit, activating T cells and possibly transporting them to the infection site. | | Grade 2–3 injection site reaction consisting of local erythema and warmness | Lymphopenia |
|  |  | A double-blind, randomized, placebo-controlled trial | NCT03821038[55] | 21(CYT107=15; placebo =6) | Change in ALC at day 29 between the placebo and treatment groups. | Intravenous CYT107 restored sepsis-induced lymphopenia, whereas intramuscular is superior. | | Intravenous CYT107 administration probably induce transient respiratory distress that ultimately led to the early termination of the study. | Lymphopenia |
| Nivolumab | A fully human immunoglobulin G4 (IgG4) anti-PD-1 antibody | A Multicenter, Open-Label Phase 1/2 Study | JapicCTI-173600[56] | 13(480mg=15;960mg=16) | Assess safety outcomes including monitoring of AEs, serious AEs, and immune-related AEs | Nivolumab at 480 mg and 960 mg increased ALC and monocyte human leukocyte antigen-DR subtype transcript levels in sepsis patients. 960 mg of nivolumab was well-tolerated and maintained blood levels. | | Organizing pneumonia (Grade 1 to Grade 2); Grade 1 C-reactive protein increase; Grade 4 alanine transaminase increase; Grade 3 aspartate transaminase increase; Grade 1 alkaline phosphatase increase; Grade 2 rash; Grade 2 pruritus; and Grade 1 hypothyroidism | N |
|  |  | A randomized, double-blind, parallel-group, Phase 1b study | NCT02960854[57] | 31(480mg=5;960mg=8) | Assess safety and PK parameters | Nivolumab did not cause a "cytokine storm," and the PK profile maintained RO > 90% for 28 days. | | Anemia, pyrexia, worsening hypotension, pleural effusion, diarrhea, and hypernatremia | N |
| BMS-936559 | Fully human IgG4 monoclonal antibody that inhibits binding of PD-L1 to both PD-1 and CD80 | A Phase 1b Randomized, Placebo-Controlled, Single Ascending Dose Study | NCT02576457[58] | 24(placebo=4; BMS-936559=20) | Death and AEs | BMS-936559 was well-tolerated. Anti-PD-L1 target engagement and mHLA-DR expression increased dose-dependently. | | Hypotension, diarrhea and delirium, anemia, increased leaps and pleural effusion, and decreased weight, hypokalemia, and malnutrition. | N |
| Immunoglobulin | Recognize and bind antigens and be able to activate the complement system and immune cells of the innate immune system. | A prospective randomised controlled study | N[59] | 33(placebo=17; IgM-enriched immunoglobulin=16) | Investigated the effects of polyclonal immunoglobulin treatment in patients with acute respiratory failure due to septic shock. | The IgM-enriched immunoglobulin preparation did not ameliorate organ dysfunction in comparison to standard sepsis therapy. | | N | N |
|  |  | A double-blind, randomized, controlled trial | ISRCTN94984750. opens in new tab[60] | 3493(placebo=1734; polyvalent IgG immune globulin=1759) | Death or major disability at the age of 2 years | Polyvalent IgG immune globulin was not associated with significant differences in the risk of severe complications, mortality, or severe disability at age 2 in neonates with suspected or confirmed sepsis. | | There was no significant difference in the overall incidence of adverse events. | N |
|  |  | A prospective, randomized, double-blinded and placebo-controlled trial | NCT01867645[61] | 38(placebo=19; IgM-enriched IVIG=19) | CIPNM severity sum score on Day 14 | Early therapy with IVIG did not mitigate CIPNM in critically ill patients with MOF and SIRS/sepsis, and after an intermediate analysis the study was terminated early due to futility in meeting the primary goal. | | N | N |
|  |  | A prospective, observational, randomized study | N[62] | 272(standard antibiotic=136; standard antibiotic plus pentaglobin=19) | Hospitalization length, mortality rate, and composite end-points | Neonatal sepsis treated with Pentaglobin (IgM-enriched intravenous immunoglobulin [IgM-IVIG] product) as an adjunct significantly reduced mortality and length of hospital stay. | | N | N |
|  |  | A retrospective cohort study | N[63] | 80(low group with serum IgG levels <829 mg/dL=40; high group with serum IgG levels ≥829 mg/dL=40) | 28-day survival probability | Regardless of serum IgG levels, there were no significant differences in the probability of survival at 28 days, length of artificial ventilation and ICU stay in IVIG-treated patients with septic shock. | | N | N |
|  |  | A double-blinded randomised controlled trial | N[64] | 80(placebo=40; immunoglobulin=40) | Duration of hospital stay in days | Intravenous immunoglobulin did not shorten the duration of hospital stay. | | N | N |
| Mesenchymal stem cell | Modulate inflammation, enhance tissue repair and pathogen clearance | An open-label phase I, dose escalation clinical trial | NCT02421484[65] | 30 (Observational =21; 0.3, 1.0, and 3.0 million cells/kg n=3 in each group) | The safety and tolerability of MSCs in septic shock | Patients with septic shock may receive up to 3 million cells/kg (250 million cells) of freshly cultured allogenic bone marrow-derived MSCs without adverse effects. | | No adverse  events were noted that were deemed related to the study. | N |
|  |  | A single-center, open-label, dose-escalation phase 1 clinical trial | ChiCTR-TRC-14005094[66] | 15 (1 × 106, 2 × 106, and 3 × 106 cells/kg n=5 in each group) | The incidence of infusion-associated events and serious adverse events. | Single intravenous injection of allogeneic MSCs (at a dose of 3 × 106 cells/kg) is safe and well tolerated in patients with severe sepsis. | | No adverse  events were noted that were deemed related to the study. | N |
|  |  | A randomized, Single-blind, parallel group, placebo controlled trial. | NCT02328612[67] | 32(placebo=8, 0.25 × 106, 1 × 106, or 4 × 106 cells/kg n=8 in each group) | Clinical signs & symptoms, leukocyte activation, cytokine release, activation of coagulation and vascular endothelium, genome wide mRNA expression profiles and ex vivo responsiveness of blood leukocyte. | The high ASC dose had multiple pro-inflammatory, anti-inflammatory and pro-coagulant effects during human endotoxemia. | | Throat irritation and pruritus | N |
| HBD/DIC: hepatobiliary dysfunction and disseminated intravascular coagulation; SOFA score: sequential organ failure assessment; TEAEs: treatment-emergent adverse events; TLR4: toll like receptor 4; PMX HP: MITT: modified intent-to-treat; RAI: relative adrenal insufficiency; ARDS: respiratory distress syndrome; polymyxin B hemoperfusion; UIT: ulinastatin; VAP: ventilator-associated pneumonia; CVVH: veno-venous hemofiltration; G-CSF: granulocyte colony-stimulating factor; GM-CSF: granulocyte-macrophage colony-stimulating factor; IFN-γ: interferon-gamma; PK; pharmacokinetics; RO: receptor occupancy; ALC: absolute lymphocyte count; AEs: adverse events; IVIG: intravenous immunoglobulins; CIPNM: Critical illness polyneuropathy and/or myopathy; MSCs: mesenchymal stem cell; N:not reported | | | | | | | | | |

**References**

1. Shakoory B, Carcillo J A, Chatham W W, Amdur R L, Zhao H, Dinarello C A, et al. Interleukin-1 Receptor Blockade Is Associated With Reduced Mortality in Sepsis Patients With Features of Macrophage Activation Syndrome: Reanalysis of a Prior Phase III Trial. Crit Care Med. 2016; **44**: 275-81. doi:10.1097/ccm.0000000000001402.

2. Leventogiannis K, Kyriazopoulou E, Antonakos N, Kotsaki A, Tsangaris I, Markopoulou D, et al. Toward personalized immunotherapy in sepsis: The PROVIDE randomized clinical trial. Cell Reports. Medicine. 2022; **3**: 100817. doi:10.1016/j.xcrm.2022.100817.

3. Morris P E, Zeno B, Bernard A C, Huang X, Das S, Edeki T, et al. A placebo-controlled, double-blind, dose-escalation study to assess the safety, tolerability and pharmacokinetics/pharmacodynamics of single and multiple intravenous infusions of AZD9773 in patients with severe sepsis and septic shock. Crit Care. 2012; **16**: R31. doi:10.1186/cc11203.

4. Aikawa N, Takahashi T, Fujimi S, Yokoyama T, Yoshihara K, Ikeda T, et al. A Phase II study of polyclonal anti-TNF-α (AZD9773) in Japanese patients with severe sepsis and/or septic shock. J Infect Chemother. 2013; **19**: 931-40. doi:10.1007/s10156-013-0612-y.

5. Bernard G R, Francois B, Mira J-P, Vincent J-L, Dellinger R P, Russell J A, et al. Evaluating the efficacy and safety of two doses of the polyclonal anti-tumor necrosis factor-α fragment antibody AZD9773 in adult patients with severe sepsis and/or septic shock: randomized, double-blind, placebo-controlled phase IIb study*. Critical Care Medicine. 2014; **42**: 504-511. doi:10.1097/CCM.0000000000000043.

6. Tidswell M, Tillis W, Larosa S P, Lynn M, Wittek A E, Kao R, et al. Phase 2 trial of eritoran tetrasodium (E5564), a toll-like receptor 4 antagonist, in patients with severe sepsis. Crit Care Med. 2010; **38**: 72-83. doi:10.1097/CCM.0b013e3181b07b78.

7. Rice T W, Wheeler A P, Bernard G R, Vincent J L, Angus D C, Aikawa N, et al. A randomized, double-blind, placebo-controlled trial of TAK-242 for the treatment of severe sepsis. Crit Care Med. 2010; **38**: 1685-94. doi:10.1097/CCM.0b013e3181e7c5c9.

8. Opal S M, Laterre P F, Francois B, LaRosa S P, Angus D C, Mira J P, et al. Effect of eritoran, an antagonist of MD2-TLR4, on mortality in patients with severe sepsis: the ACCESS randomized trial. Jama. 2013; **309**: 1154-62. doi:10.1001/jama.2013.2194.

9. Arabi Y M, Aljumah A, Dabbagh O, Tamim H M, Rishu A H, Al-Abdulkareem A, et al. Low-dose hydrocortisone in patients with cirrhosis and septic shock: a randomized controlled trial. Cmaj. 2010; **182**: 1971-7. doi:10.1503/cmaj.090707.

10. Huh J W, Choi H S, Lim C M, Koh Y, Oh Y M, Shim T S, et al. Low-dose hydrocortisone treatment for patients with septic shock: a pilot study comparing 3days with 7days. Respirology. 2011; **16**: 1088-95. doi:10.1111/j.1440-1843.2011.02018.x.

11. Gordon A C, Mason A J, Perkins G D, Stotz M, Terblanche M, Ashby D, et al. The interaction of vasopressin and corticosteroids in septic shock: a pilot randomized controlled trial. Crit Care Med. 2014; **42**: 1325-33. doi:10.1097/ccm.0000000000000212.

12. Gordon A C, Mason A J, Thirunavukkarasu N, Perkins G D, Cecconi M, Cepkova M, et al. Effect of Early Vasopressin vs Norepinephrine on Kidney Failure in Patients With Septic Shock: The VANISH Randomized Clinical Trial. Jama. 2016; **316**: 509-18. doi:10.1001/jama.2016.10485.

13. Tongyoo S, Permpikul C, Mongkolpun W, Vattanavanit V, Udompanturak S, Kocak M, et al. Hydrocortisone treatment in early sepsis-associated acute respiratory distress syndrome: results of a randomized controlled trial. Crit Care. 2016; **20**: 329. doi:10.1186/s13054-016-1511-2.

14. Hyvernat H, Barel R, Gentilhomme A, Césari-Giordani J F, Freche A, Kaidomar M, et al. Effects of Increasing Hydrocortisone to 300 mg Per Day in the Treatment of Septic Shock: a Pilot Study. Shock. 2016; **46**: 498-505. doi:10.1097/shk.0000000000000665.

15. Keh D, Trips E, Marx G, Wirtz S P, Abduljawwad E, Bercker S, et al. Effect of Hydrocortisone on Development of Shock Among Patients With Severe Sepsis: The HYPRESS Randomized Clinical Trial. Jama. 2016; **316**: 1775-1785. doi:10.1001/jama.2016.14799.

16. Menon K, McNally D, O'Hearn K, Acharya A, Wong H R, Lawson M, et al. A Randomized Controlled Trial of Corticosteroids in Pediatric Septic Shock: A Pilot Feasibility Study. Pediatr Crit Care Med. 2017; **18**: 505-512. doi:10.1097/pcc.0000000000001121.

17. Ngaosuwan K, Ounchokdee K,Chalermchai T. Clinical Outcomes of Minimized Hydrocortisone Dosage of 100 Mg/Day on Lower Occurrence of Hyperglycemia in Septic Shock Patients. Shock. 2018; **50**: 280-285. doi:10.1097/shk.0000000000001061.

18. Venkatesh B, Finfer S, Cohen J, Rajbhandari D, Arabi Y, Bellomo R, et al. Adjunctive Glucocorticoid Therapy in Patients with Septic Shock. N Engl J Med. 2018; **378**: 797-808. doi:10.1056/NEJMoa1705835.

19. Annane D, Renault A, Brun-Buisson C, Megarbane B, Quenot J P, Siami S, et al. Hydrocortisone plus Fludrocortisone for Adults with Septic Shock. N Engl J Med. 2018; **378**: 809-818. doi:10.1056/NEJMoa1705716.

20. Tilouche N, Jaoued O, Ali H B S, Gharbi R, Fekih Hassen M,Elatrous S. Comparison Between Continuous and Intermittent Administration of Hydrocortisone During Septic Shock: A Randomized Controlled Clinical Trial. Shock. 2019; **52**: 481-486. doi:10.1097/shk.0000000000001316.

21. Kang J, Han M, Hong S B, Lim C M, Koh Y,Huh J W. Effect of adjunctive corticosteroid on 28-day mortality in neutropenic patients with septic shock. Ann Hematol. 2019; **98**: 2311-2318. doi:10.1007/s00277-019-03785-w.

22. Fujii T, Luethi N, Young P J, Frei D R, Eastwood G M, French C J, et al. Effect of Vitamin C, Hydrocortisone, and Thiamine vs Hydrocortisone Alone on Time Alive and Free of Vasopressor Support Among Patients With Septic Shock: The VITAMINS Randomized Clinical Trial. Jama. 2020; **323**: 423-431. doi:10.1001/jama.2019.22176.

23. Iglesias J, Vassallo A V, Patel V V, Sullivan J B, Cavanaugh J,Elbaga Y. Outcomes of Metabolic Resuscitation Using Ascorbic Acid, Thiamine, and Glucocorticoids in the Early Treatment of Sepsis: The ORANGES Trial. Chest. 2020; **158**: 164-173. doi:10.1016/j.chest.2020.02.049.

24. Moskowitz A, Huang D T, Hou P C, Gong J, Doshi P B, Grossestreuer A V, et al. Effect of Ascorbic Acid, Corticosteroids, and Thiamine on Organ Injury in Septic Shock: The ACTS Randomized Clinical Trial. Jama. 2020; **324**: 642-650. doi:10.1001/jama.2020.11946.

25. Sevransky J E, Rothman R E, Hager D N, Bernard G R, Brown S M, Buchman T G, et al. Effect of Vitamin C, Thiamine, and Hydrocortisone on Ventilator- and Vasopressor-Free Days in Patients With Sepsis: The VICTAS Randomized Clinical Trial. Jama. 2021; **325**: 742-750. doi:10.1001/jama.2020.24505.

26. Hussein A A, Sabry N A, Abdalla M S,Farid S F. A prospective, randomised clinical study comparing triple therapy regimen to hydrocortisone monotherapy in reducing mortality in septic shock patients. Int J Clin Pract. 2021; **75**: e14376. doi:10.1111/ijcp.14376.

27. Lyu Q Q, Zheng R Q, Chen Q H, Yu J Q, Shao J,Gu X H. Early administration of hydrocortisone, vitamin C, and thiamine in adult patients with septic shock: a randomized controlled clinical trial. Crit Care. 2022; **26**: 295. doi:10.1186/s13054-022-04175-x.

28. Takeyama N, Noguchi H, Hirakawa A, Kano H, Morino K, Obata T, et al. Time to initiation of treatment with polymyxin B cartridge hemoperfusion in septic shock patients. Blood Purif. 2012; **33**: 252-6. doi:10.1159/000336341.

29. Payen D M, Guilhot J, Launey Y, Lukaszewicz A C, Kaaki M, Veber B, et al. Early use of polymyxin B hemoperfusion in patients with septic shock due to peritonitis: a multicenter randomized control trial. Intensive Care Med. 2015; **41**: 975-84. doi:10.1007/s00134-015-3751-z.

30. Saito N, Sugiyama K, Ohnuma T, Kanemura T, Nasu M, Yoshidomi Y, et al. Efficacy of polymyxin B-immobilized fiber hemoperfusion for patients with septic shock caused by Gram-negative bacillus infection. PLoS One. 2017; **12**: e0173633. doi:10.1371/journal.pone.0173633.

31. Yaroustovsky M, Abramyan M, Komardina E, Nazarova H, Popov D, Plyushch M, et al. Selective LPS Adsorption Using Polymyxin B-Immobilized Fiber Cartridges in Sepsis Patients Following Cardiac Surgery. Shock. 2018; **49**: 658-666. doi:10.1097/shk.0000000000001016.

32. Dellinger R P, Bagshaw S M, Antonelli M, Foster D M, Klein D J, Marshall J C, et al. Effect of Targeted Polymyxin B Hemoperfusion on 28-Day Mortality in Patients With Septic Shock and Elevated Endotoxin Level: The EUPHRATES Randomized Clinical Trial. Jama. 2018; **320**: 1455-1463. doi:10.1001/jama.2018.14618.

33. Srisawat N, Tungsanga S, Lumlertgul N, Komaenthammasophon C, Peerapornratana S, Thamrongsat N, et al. The effect of polymyxin B hemoperfusion on modulation of human leukocyte antigen DR in severe sepsis patients. Crit Care. 2018; **22**: 279. doi:10.1186/s13054-018-2077-y.

34. Nakata H, Yamakawa K, Kabata D, Umemura Y, Ogura H, Gando S, et al. Identifying Septic Shock Populations Benefitting From Polymyxin B Hemoperfusion: A Prospective Cohort Study Incorporating a Restricted Cubic Spline Regression Model. Shock. 2020; **54**: 667-674. doi:10.1097/shk.0000000000001533.

35. Karnad D R, Bhadade R, Verma P K, Moulick N D, Daga M K, Chafekar N D, et al. Intravenous administration of ulinastatin (human urinary trypsin inhibitor) in severe sepsis: a multicenter randomized controlled study. Intensive Care Med. 2014; **40**: 830-8. doi:10.1007/s00134-014-3278-8.

36. Meng F, Du C, Zhang Y, Wang S, Zhou Q, Wu L, et al. Protective effect of rhubarb combined with ulinastatin for patients with sepsis. Medicine (Baltimore). 2020; **99**: e18895. doi:10.1097/md.0000000000018895.

37. Zhang H, Wei L, Zhao G, Liu S, Zhang Z, Zhang J, et al. Protective effect of Xuebijing injection on myocardial injury in patients with sepsis: a randomized clinical trial. J Tradit Chin Med. 2016; **36**: 706-10. doi:10.1016/s0254-6272(17)30003-1.

38. Liu Y, Wang B, Zhang Q, Zhao Y,Wang X. A Case-Control Study of Continuous Veno-Venous Hemofiltration Combined with Xuebijing Injection in the Treatment of Severe Sepsis. Contrast Media Mol Imaging. 2022; **2022**: 7884508. doi:10.1155/2022/7884508.

39. Liu S, Yao C, Xie J, Liu H, Wang H, Lin Z, et al. Effect of an Herbal-Based Injection on 28-Day Mortality in Patients With Sepsis: The EXIT-SEP Randomized Clinical Trial. JAMA Intern Med. 2023. doi:10.1001/jamainternmed.2023.0780.

40. Giamarellos-Bourboulis E J, Mylona V, Antonopoulou A, Tsangaris I, Koutelidakis I, Marioli A, et al. Effect of clarithromycin in patients with suspected Gram-negative sepsis: results of a randomized controlled trial. J Antimicrob Chemother. 2014; **69**: 1111-8. doi:10.1093/jac/dkt475.

41. Tsaganos T, Raftogiannis M, Pratikaki M, Christodoulou S, Kotanidou A, Papadomichelakis E, et al. Clarithromycin Leads to Long-Term Survival and Cost Benefit in Ventilator-Associated Pneumonia and Sepsis. Antimicrob Agents Chemother. 2016; **60**: 3640-6. doi:10.1128/aac.02974-15.

42. Karakike E, Scicluna B P, Roumpoutsou M, Mitrou I, Karampela N, Karageorgos A, et al. Effect of intravenous clarithromycin in patients with sepsis, respiratory and multiple organ dysfunction syndrome: a randomized clinical trial. Crit Care. 2022; **26**: 183. doi:10.1186/s13054-022-04055-4.

43. Gathwala G, Walia M, Bala H,Singh S. Recombinant human granulocyte colony-stimulating factor in preterm neonates with sepsis and relative neutropenia: a randomized, single-blind, non-placebo-controlled trial. J Trop Pediatr. 2012; **58**: 12-8. doi:10.1093/tropej/fmr012.

44. El-Ganzoury M M, El-Farrash R A, Saad A A, Mohamed A G,El-Sherbini I G. In vivo effect of recombinant human granulocyte colony-stimulating factor on neutrophilic expression of CD11b in septic neonates: a randomized controlled trial. Pediatr Hematol Oncol. 2012; **29**: 272-84. doi:10.3109/08880018.2011.644880.

45. Aktaş D, Demirel B, Gürsoy T,Ovalı F. A randomized case-controlled study of recombinant human granulocyte colony stimulating factor for the treatment of sepsis in preterm neutropenic infants. Pediatr Neonatol. 2015; **56**: 171-5. doi:10.1016/j.pedneo.2014.06.007.

46. Marlow N, Morris T, Brocklehurst P, Carr R, Cowan F M, Patel N, et al. A randomised trial of granulocyte-macrophage colony-stimulating factor for neonatal sepsis: outcomes at 2 years. Arch Dis Child Fetal Neonatal Ed. 2013; **98**: F46-53. doi:10.1136/fetalneonatal-2011-301470.

47. Marlow N, Morris T, Brocklehurst P, Carr R, Cowan F, Patel N, et al. A randomised trial of granulocyte-macrophage colony-stimulating factor for neonatal sepsis: childhood outcomes at 5 years. Arch Dis Child Fetal Neonatal Ed. 2015; **100**: F320-6. doi:10.1136/archdischild-2014-307410.

48. Hall M W, Knatz N L, Vetterly C, Tomarello S, Wewers M D, Volk H D, et al. Immunoparalysis and nosocomial infection in children with multiple organ dysfunction syndrome. Intensive Care Med. 2011; **37**: 525-32. doi:10.1007/s00134-010-2088-x.

49. Leentjens J, Kox M, Koch R M, Preijers F, Joosten L A B, van der Hoeven J G, et al. Reversal of immunoparalysis in humans in vivo: a double-blind, placebo-controlled, randomized pilot study. American Journal of Respiratory and Critical Care Medicine. 2012; **186**: 838-845. doi:10.1164/rccm.201204-0645OC.

50. Wu J, Zhou L, Liu J, Ma G, Kou Q, He Z, et al. The efficacy of thymosin alpha 1 for severe sepsis (ETASS): a multicenter, single-blind, randomized and controlled trial. Crit Care. 2013; **17**: R8. doi:10.1186/cc11932.

51. Bai L, Qiu X, Ding X, Bai X, Huang W, Yang L, et al. Value of Thymosin α1 Combined With Blood Purification to Increase Successful Rescues of Shock Patients. Altern Ther Health Med. 2022; **28**: 146-152.

52. Delsing C E, Gresnigt M S, Leentjens J, Preijers F, Frager F A, Kox M, et al. Interferon-gamma as adjunctive immunotherapy for invasive fungal infections: a case series. BMC Infect Dis. 2014; **14**: 166. doi:10.1186/1471-2334-14-166.

53. Payen D, Faivre V, Miatello J, Leentjens J, Brumpt C, Tissières P, et al. Multicentric experience with interferon gamma therapy in sepsis induced immunosuppression. A case series. BMC Infectious Diseases. 2019; **19**: 931. doi:10.1186/s12879-019-4526-x.

54. Francois B, Jeannet R, Daix T, Walton A H, Shotwell M S, Unsinger J, et al. Interleukin-7 restores lymphocytes in septic shock: the IRIS-7 randomized clinical trial. JCI Insight. 2018; **3**. doi:10.1172/jci.insight.98960.

55. Daix T, Mathonnet A, Brakenridge S, Dequin P F, Mira J P, Berbille F, et al. Intravenously administered interleukin-7 to reverse lymphopenia in patients with septic shock: a double-blind, randomized, placebo-controlled trial. Ann Intensive Care. 2023; **13**: 17. doi:10.1186/s13613-023-01109-w.

56. Watanabe E, Nishida O, Kakihana Y, Odani M, Okamura T, Harada T, et al. Pharmacokinetics, Pharmacodynamics, and Safety of Nivolumab in Patients With Sepsis-Induced Immunosuppression: A Multicenter, Open-Label Phase 1/2 Study. Shock. 2020; **53**: 686-694. doi:10.1097/shk.0000000000001443.

57. Hotchkiss R S, Colston E, Yende S, Crouser E D, Martin G S, Albertson T, et al. Immune checkpoint inhibition in sepsis: a Phase 1b randomized study to evaluate the safety, tolerability, pharmacokinetics, and pharmacodynamics of nivolumab. Intensive Care Med. 2019; **45**: 1360-1371. doi:10.1007/s00134-019-05704-z.

58. Hotchkiss R S, Colston E, Yende S, Angus D C, Moldawer L L, Crouser E D, et al. Immune Checkpoint Inhibition in Sepsis: A Phase 1b Randomized, Placebo-Controlled, Single Ascending Dose Study of Antiprogrammed Cell Death-Ligand 1 Antibody (BMS-936559). Crit Care Med. 2019; **47**: 632-642. doi:10.1097/ccm.0000000000003685.

59. Toth I, Mikor A, Leiner T, Molnar Z, Bogar L,Szakmany T. Effects of IgM-enriched immunoglobulin therapy in septic-shock-induced multiple organ failure: pilot study. J Anesth. 2013; **27**: 618-22. doi:10.1007/s00540-012-1553-9.

60. Brocklehurst P, Farrell B, King A, Juszczak E, Darlow B, Haque K, et al. Treatment of neonatal sepsis with intravenous immune globulin. N Engl J Med. 2011; **365**: 1201-11. doi:10.1056/NEJMoa1100441.

61. Brunner R, Rinner W, Haberler C, Kitzberger R, Sycha T, Herkner H, et al. Early treatment with IgM-enriched intravenous immunoglobulin does not mitigate critical illness polyneuropathy and/or myopathy in patients with multiple organ failure and SIRS/sepsis: a prospective, randomized, placebo-controlled, double-blinded trial. Crit Care. 2013; **17**: R213. doi:10.1186/cc13028.

62. Nassir K F, Al-Saddi Y I, Abbas H M, Al Khames Aga Q A, Al Khames Aga L A,Oudah A A. Pentaglobin (immunoglobulin M-enriched immunoglobulin) as adjuvant therapy for premature and very low-birth-weight neonates with sepsis. Indian J Pharmacol. 2021; **53**: 364-370. doi:10.4103/ijp.ijp_881_20.

63. Goto K, Yasuda N,Sato Y. Effects of low-dose intravenous immunoglobulin as the adjunctive therapy in septic shock patients with and without hypogammaglobulinemia: a retrospective cohort study. Ann Palliat Med. 2022; **11**: 2600-2608. doi:10.21037/apm-21-3694.

64. Rizvi M Q, Singh M V, Mishra N, Shrivastava A, Maurya M,Siddiqui S A. Intravenous immunoglobulin in the management of neonatal sepsis: A randomised controlled trial. Trop Doct. 2023; **53**: 222-226. doi:10.1177/00494755221138689.

65. McIntyre L A, Stewart D J, Mei S H J, Courtman D, Watpool I, Granton J, et al. Cellular Immunotherapy for Septic Shock. A Phase I Clinical Trial. Am J Respir Crit Care Med. 2018; **197**: 337-347. doi:10.1164/rccm.201705-1006OC.

66. He X, Ai S, Guo W, Yang Y, Wang Z, Jiang D, et al. Umbilical cord-derived mesenchymal stem (stromal) cells for treatment of severe sepsis: aphase 1 clinical trial. Transl Res. 2018; **199**: 52-61. doi:10.1016/j.trsl.2018.04.006.

67. Perlee D, van Vught L A, Scicluna B P, Maag A, Lutter R, Kemper E M, et al. Intravenous Infusion of Human Adipose Mesenchymal Stem Cells Modifies the Host Response to Lipopolysaccharide in Humans: A Randomized, Single-Blind, Parallel Group, Placebo Controlled Trial. Stem Cells. 2018; **36**: 1778-1788. doi:10.1002/stem.2891.
